# Sickness in the Rebel Army

**Published:** 1861-11

**Authors:** 


*Sickness in the Rebel Army.*— A recent copy of the Charleston *Mercury* contains an editorial article, in which it speaks of the bad food furnished by the Commissarat at Richmond. It speaks of "fifteen thousand now lying sick, scattered all around Manassas." It adds that the Commissary Department "furnish raw wheat flour, and leave the poor soldiers to work it into dough, which has proved more fatal to the army than Yankee rifles and cannon." In the same paper it is also stated that "the number of disabled volunteers in Richmond increases with each day's arrival from Manassas. On Sunday, the Central cars brought down 100 of the sick, who were immediately distributed in the different hospitals. An arrival on Monday morning added 150 patients to the list."—*Medical and Surgical Reporter.*

